# Revealing instances of coordination among multiple cortical areas

**DOI:** 10.1007/s00422-013-0574-2

**Published:** 2013-11-01

**Authors:** M. Abeles

**Affiliations:** 1 Gonda Brain Research Center, Bar-Ilan University, Max and Anna Webb Str., 52900 Ramat Gan, Israel; 2 The Hebrew University of Jerusalem, Jerusalem, Israel

**Keywords:** MEG, Binding, Cortical current dipoles, Higher brain functions

## Abstract

Cognitive functions must involve interactions between several (perhaps many) cortical regions. The instances of such interactions may not be tightly time locked to any external cue. Thus averaging over repeated trials of brain activity or its spectrograms may miss these instances. Here, coordinated activity among multiple cortical locations is revealed in ongoing activity with millisecond accuracy without the need for averaging over time or frequencies. This is based on reconstructions of the cortical current dipole amplitudes at multiple points from MEG recordings. In these current dipole traces, instances of brief activity undulations (BAUs) are automatically detected and used to reveal where and when cortical points interact. The article shows that these BAUs truly represent the reorganization of activity at the cortex and are strongly connected to behavior.

## Introduction

For over 30 years, the mechanisms by which neuronal activities become bound to generate a percept or any other complex mental entity is being discussed, see for example, some of the behavioral, electrophysiological, and theoretical deliberations in (Triesman and Gelade 1980; Engel and Singer 2001; von der Malsburg 1999). The study of which areas in the human cortex become coordinated and at what time is carried out mostly by averaging over time and frequency of macroscopic recordings (EEG, ECoG, and MEG). Such methods do not allow for detecting the precise instance at which cortical areas start to interact.

Here, we take advantage of the anatomical finding that cortico-cortical connections are excitatory, whereas inhibition is local (Abeles 1991; Braitenberg and Schüz 1998). Therefore, it can be assumed that when two or more cortical regions become engaged in processing the same data, there will be an increase in activity in both areas that will be then quenched by the local inhibition. This quenching may restore activity to baseline or may yield short oscillatory bursts of periodic changes in the level of activity (spindles). We show here that such brief activity undulations (BAUs) exist abundantly in cortical current dipoles (CCDs) and involve different brain regions during different behavioral tasks. This makes it feasible to study ongoing brain activity without having to average over repetitions of the same event or for time or spectralaveraging.

The analysis is based on the following hypotheses:

1. Mental activities are generated by coordinated activity in many brain regions.
2. When two regions start to coordinate their activity, there is a brief increase in activity in both.
3. Such brief increases may be detected in recordings of electrical activity of a cortical region.
4. When the same mental task is repeated frequently, the same regions become coordinated in the same manner.

## Methods

### Recordings

MEG was recorded on a Magnus 3,600 machine equipped with 248 magnetometers and 23 reference channels. Data were filtered at 1–800 Hz and sampled at 2,034.5/s. Power line, video, heartbeat, and vibration artifacts were cleaned offline by the methods described in Tal and Abeles (2013).

The subject was lying in a supine position to reduce head position drifts during recording and to make sure that the brain’s position in the skull was as close as possible to that in the MRI. Head shape was measured with Polhemus Fastrak and 3 fiduciary points were marked. Coils were attached to these points. The coils and their position in the MEG hood were measured. These measurements were used to position the brain’s MRI image in the MEG’s hood as accurately as possible. Finally, fine adjustments were made manually. The brain’s hull was extracted by Analysis of Functional NeuroImages (AFNI) [http://afni.nimh.nih.gov/afni/].

The subject was instructed to engage in 7 tasks lasting 2 min each. For the first 2 min, he was instructed to relax with his eyes closed. This was designed to be a period in which he could get used to the recording situation (this period is referred to as *Relax0*). In the second period, he was asked to continue to relax for 2 min (*Relax1*). In the third period, he was asked to play with his right-hand fingers and from time to time invent a new sequence of finger motions as he pleased (*FmovR*). In the fourth period, he was asked to play with his left-hand fingers (*FmovL*). Then, he was asked to count down from 3,001 in steps of 7 (*CntDwn*). Then, he was asked to remember scenes from the last movie he had seen (*Rmmbr*). Finally, he was asked to relax again (*Relax2*). The various tasks were described before the recording session. During the recording, a one-sentence reminder of what he was expected to do was announced through the intercom every two minutes. Data from 10 s up to 110 s after the announcement were used for analysis.

### Reconstructing cortical current dipoles (CCDs)

**Fig. 1:**
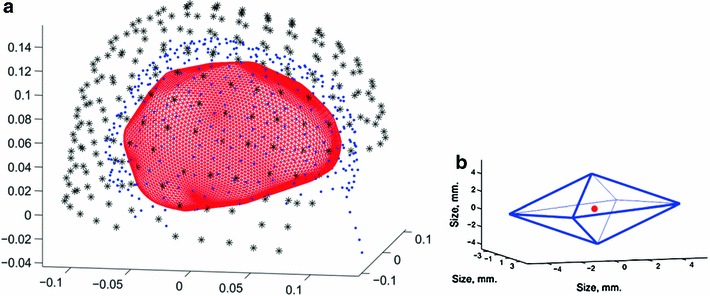
Hull & octahedron. **a** Head sensor and brain hull. The measurements on the axes are in meters. *Black stars*—location of the MEG sensors. *Blue points*—points on the head surface. *Red mesh*—the brain hull. The large distance of the sensors from the brain results in considerable similarity between the recorded signals at adjacent sensors. **b** Octahedron. *Blue*—the contours of the octahedron. *Red*—the point of interest at the center of the octahedron. Amplitudes for the current dipoles were simultaneously extracted for all 7 points (vertices + center), but only the dipole for the center was used for further analysis

Seven hundred points of interest were selected over the brain’s hull at approximately equal distances (range of distance between next neighbors 0.7–1.3 cm). Around each of these, an octahedron was constructed with its top and bottom apices perpendicular to the hull’s surface and the square mid-plan parallel to the surface. Figure 1 illustrates the hull and one such octahedron. The distance of the center point (which was on the hull’s surface) from each of the vertices was 0.8 cm and from each of the corners of the square mid-plan was 1.6 cm. The amplitude of the current dipoles at each of the 7 points (vertices + center) was evaluated simultaneously using a synthetic aperture magnetometry (SAM) method (Robinson 2004; Sekihara and Nagarajan 2008; Moiseev et al. 2011). Only the current dipole amplitudes of the center point were considered. This approach yielded current dipoles at the cortex with small correlations (Lots et al. 2013). We term each such evaluated amplitude a cortical current dipole (CCD).

To avoid points that were too distant from the MEG sensors, we only considered targets on the dorsal and lateral aspects of the hull. This yielded 532 CCD traces out of the initial 700.

After the CCD had been evaluated, they were band-limit filtered at 3–35 Hz, but retained their original sampling rate of 2,034.5. Each trace was normalized so that its mean over the entire recordings was 0 and the standard deviation was 1. This was necessary because SAM assigns larger amplitudes as a function of the depth of the source.

### Detecting brief activity undulations (BAUs)

**Fig. 2:**
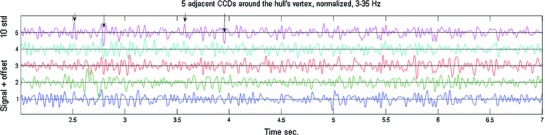
Examples of CCDs. Five seconds of activity of 5 adjacent locations at and around the hull’s vertex are illustrated. Data were sampled at 2,034.5/s and filtered at 3–35 Hz. Data were normalized by subtracting the mean and dividing by the standard deviation. *Arrows point* to instances on the top trace considered to be brief activity undulations (BAUs). Some BAUs also appear on other adjacent channels but usually not on all (e.g., the *last arrow points* to a BAU that appeared on traces 5 and 3 but not on others)

Inspection of the CCDs showed BAUs as illustrated in Fig. 2.

A few (100) of these were collected by hand and used to find an optimal template as described in the Sect. 3. Then, the template was used as described in Abeles and Goldstein (1977) to automatically detect the BAUs. For each channel, the threshold for detection was set so as to have 3–4 detections per second averaged over the entire recordings. In SAM, the best dipole direction can easily flip by 180$^{\circ }$. Because an up-pointing dipole with positive current is identical to down-pointing diploe with negative currents, the direction of the BAUs was ignored.

Brief activity undulation (BAU) timings were treated as point processes such that at the end of this process, there were 532 parallel point processes with an average rate of 3–4 Hz. We refer to each of these traces as a BAUpp.

### Statistical validation

Each 100 s of a given task was parsed at random into training and test sets. This was done by parsing the data into 10 disjoint pieces of 10 s each and then randomly selecting 5 of these to serve as a training set and the other 5 as a test set. Similarities between various features (see Sect. 3) were used to determine the degree of similarity of each test set to each of the 7 training sets (for the 7 tasks). This test set was then assigned to the task with the most similar features in its training set. This was repeated for 100 different random divisions of training and test sets to estimate the ability of the selected features to discriminate among the tasks.

Further validation was provided by mixing the original data among the tasks as follows. Each 100 s of each task was split into 98 disjoint pieces. Then, new randomly shuffled data were generated by randomly selecting 14 pieces from each task and randomly concatenating the $14\times 7$ pieces into new ‘mixed’ tasks. All analyses carried out for the real data were repeated with the randomly shuffled data. This was repeated 100 times.

## Results

### Brief activity undulations (BAUs) in cortical current dipoles (CCDs)

**Fig. 3:**
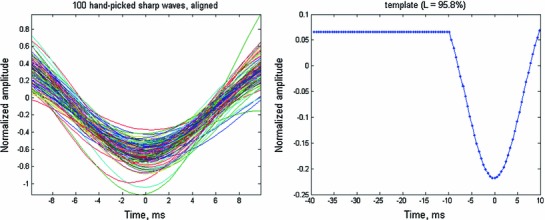
BAUs and the template. The CCDs were band-passed at 3–35 Hz. *Left*—samples of 100 BAUs identified manually. For visualization purposes, all the samples are plotted pointing downwards. All wave shapes were aligned until they had a maximal correlation with the mean. Principal component analysis was carried out on these 100 wave shapes. The first PC explained more than 96.8 % of the variance and was used as the template. *Right*—the template spans $-$10 to 10 ms. Its first value was extended by 30 ms to the *left*. Then, the mean of the whole shape was subtracted, and the shape was normalized so that the sum of all the samples squared was 1. This shape was used as a template (denoted *T* in the text). The purpose of the *straight line* inserted to the *left* was to favor the detection of the first BAU in cases where several appeared one after the other (as in a spindle)

When activity in several cortical regions becomes coordinated, we posited there would be a brief increase in activity in these regions. As predicted, there was a brief undulation in the CCDs all along the recordings. In order to detect them, we collected 100 samples of such transients, averaged them, and computed the principal components from the total covariance matrix. The first PC resembled the average and explained 96.8 % of the total variance. It was used as a template. Figure 3 depicts the 100 samples and the resulting template.

This template was used to detect similar wave shapes in the data by a procedure that treats the similarity between the recorded signal and the template as ‘signal’ and the dissimilarity as ‘noise.’ This method was described in (Abeles and Goldstein 1977). In brief, if CCD is the recorded signal and $T$ is the 101 sample-long template (Fig. 3-right), for each sample $i$, we treat the segment of the CCD spanning 101 samples ($i-50\,\mathrm{to}\,i+50$) as a vector $\tilde{V}$, and we subtract its average from it:

$$\begin{aligned} V=\tilde{V}-\frac{1}{101}\sum \limits _{j=i-50}^{i=50} {\tilde{v}_j } \end{aligned}$$

and project it on template $T$.

$$\begin{aligned} s=V\cdot {T}' \end{aligned}$$

*s* (the length of projection on $T$) is treated as the signal. The residual

$$\begin{aligned} E=V-sT \end{aligned}$$

is treated as the noise, and the signal-to-noise ratio (SNR) is given by

$$\begin{aligned} \hbox {SNR}=s^{2}/\left\| E \right\| ^{2} \end{aligned}$$

**Fig. 4:**
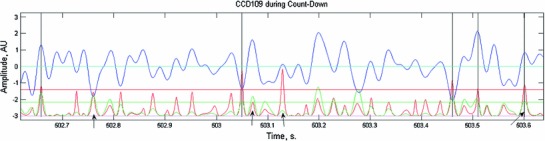
Detecting BAUs. *Blue*—a 1 s trace of the CCD at location 109 (center top of the hull). *Green*—signal squared. *Red*—SNR. Note that the peaks of the SNR are much sharper than the peaks in the CCD or the signal. Thus, they are the best marker for the position of BAUs. *Horizontal-cyan*—zero for the CCD signal. *Horizontal-magenta*—zero for the signal and SNR traces. *Horizontal green and red lines*—the thresholds for the signal and SNR, respectively. *Vertical black lines*—the time of the detected BAUs. Each of these signals is plotted in its own scale. *Arrows* from *left to right mark* examples of various cases: (1) The size of the signal is above threshold, but the wave is wider, and therefore, the SNR does not reach threshold. (2) The fit to the signal is better than for the preceding (negative) wave. However, as this wave is preceded by a large wave, the SNR is smaller and it does not meet the criteria for detection. (3) This wave had an excellent SNR (a wave of the appropriate shape preceded by an almost flat stretch). However, it is small and therefore not detected. (4) Good enough SNR and barely good enough signal size. This may count as a false positive

We selected two thresholds $T_{\mathrm{S}}$ and $T_{\mathrm{SNR}}$ and whenever $s^{2}$ was above $T_{\mathrm{S}}$ and SNR was above $T_{\mathrm{SNR}}$, we marked the time of the peak SNR as the sample at which a brief transient occurred. We term these transients BAUs (Brief Amplitude Undulations). The two thresholds were selected along the regression line of SNR versus $s^{2}$ so that over all the recorded data there would be 3–4 detections per second for each CCD. Figure 4 illustrates this procedure.

**Fig. 5:**
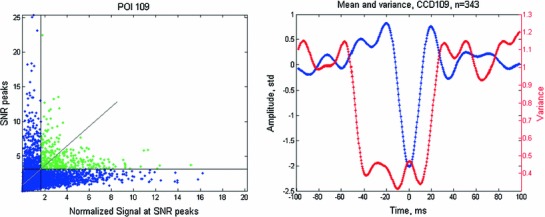
Finding short transients. Data as in Fig. 4 for 100 s of recording. The ‘signal’ and the ‘SNR’ were computed as described in the text. *Left*—the values of the SNR versus the signal squared at the peaks of SNR are plotted. The *straight oblique line* is the regression line between them. The *vertical and the horizontal lines* are the thresholds $\hbox {T}_{\mathrm{S}}$ and $\hbox {T}_{\mathrm{SNR}}$. The *green dots* are those selected for marking the times of the selected peaks. *Right*—the mean CCD at location No. 109 around 343 selected markers for short transients and its variance. *Blue*—the mean wave shape. *Red*—the mean variance around each point of the mean. (The ordinate for the variance is on the right)

The mean BAU, as seen in Fig. 5-right, reveals several points. The variance (red trace) falls off precipitously around the peak, indicating that the sharp transients in the data are not some additive feature to the ongoing activity, but rather replace much of the activity which is not related to the transient. The mean around the BAU shows small periodic undulations in the beta to gamma range. This implies that only a few of the detected BAUS were in the midst of a gamma (or beta) burst of oscillations. Thus, with this template, our method reveals mostly isolated brief transients.

### Relation to behavioral task

To validate that our CCDs and the BAUs were related to behavior, we tested for some specific relations between the BAUs and behavior. CCDs were estimated for a session in which the subject was lying supine in the MEG and executing mental tasks, as explained in the Sect. 2. All CCDs were filtered to 3–35 Hz. BAUs were detected in these CCDs for each of 532 locations in each task.

**Fig. 6:**
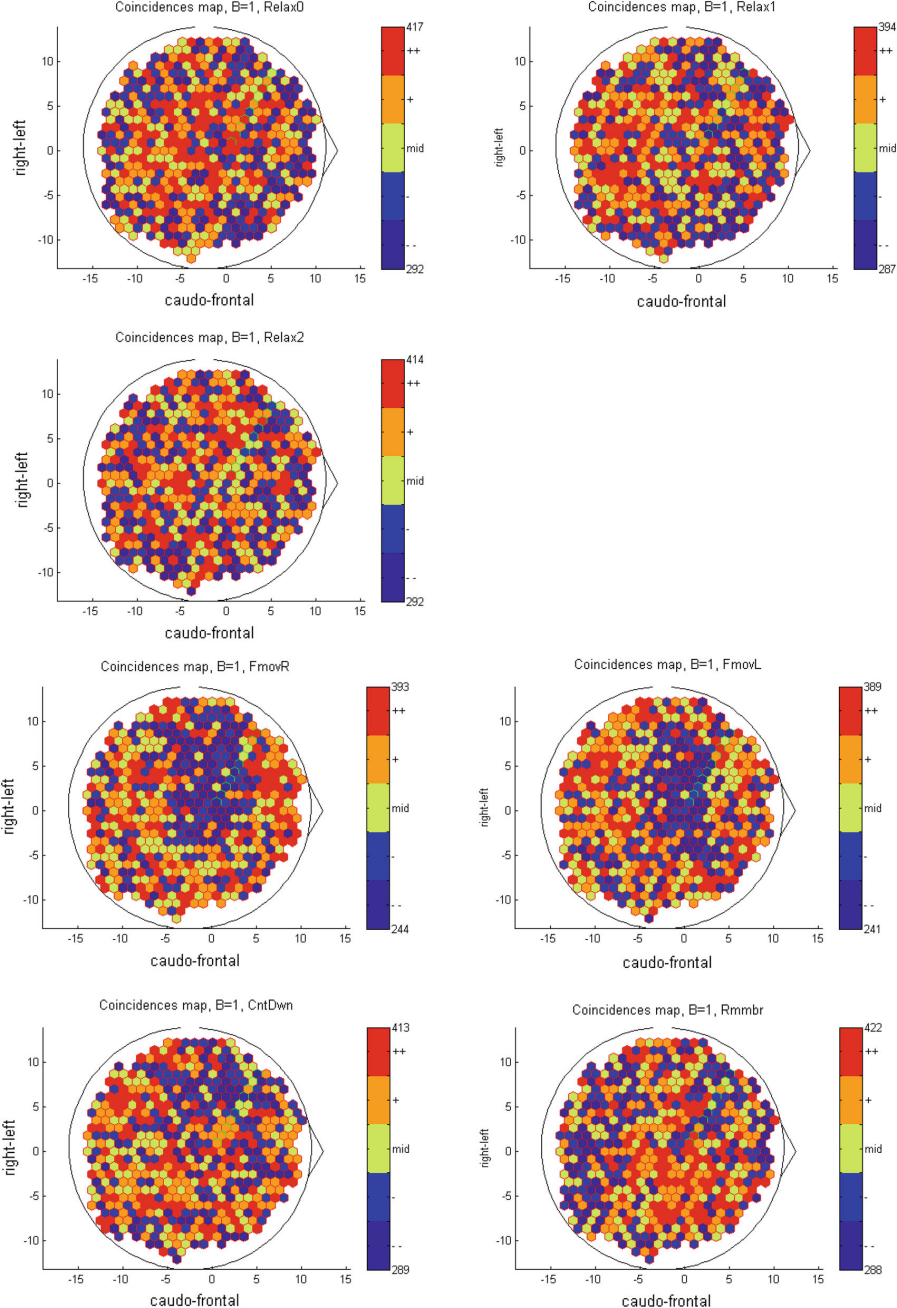
BAU-density maps. The maps area is a flat mount of the dorsal, lateral, and posterior cortex on which the CCDs were estimated. As the posterior parts are larger and contain more CCDs, the vertex of the head is shifted forward to the (0, 0) coordinates. To facilitate evaluation, the densities of each map were quantized to 5 quantiles, each containing one-fifth of the CCDs. *Top two rows*—for the three resting states. *Middle line*—for the finger motion and *bottom line*—for abstract mental activities

We treated the times of occurrence of the BAUs as a stochastic point process (like spike trains in microelectrode recordings). In this way, we obtained 532 parallel point processes organized in 7 groups of 100 s long each. By counting how many BAUs occurred in each CCD during each task, we obtained 7 BAU-density maps. These are illustrated in Fig. 6. The 3 resting states are on the top part and the states where mental activity was directed are depicted in the bottom part.

The maps are not identical and rather contain many small differences in many scattered locations. It is not clear how many of these differences are truly due to differences in mental activity, and how many are due to random fluctuations.

**Fig. 7:**
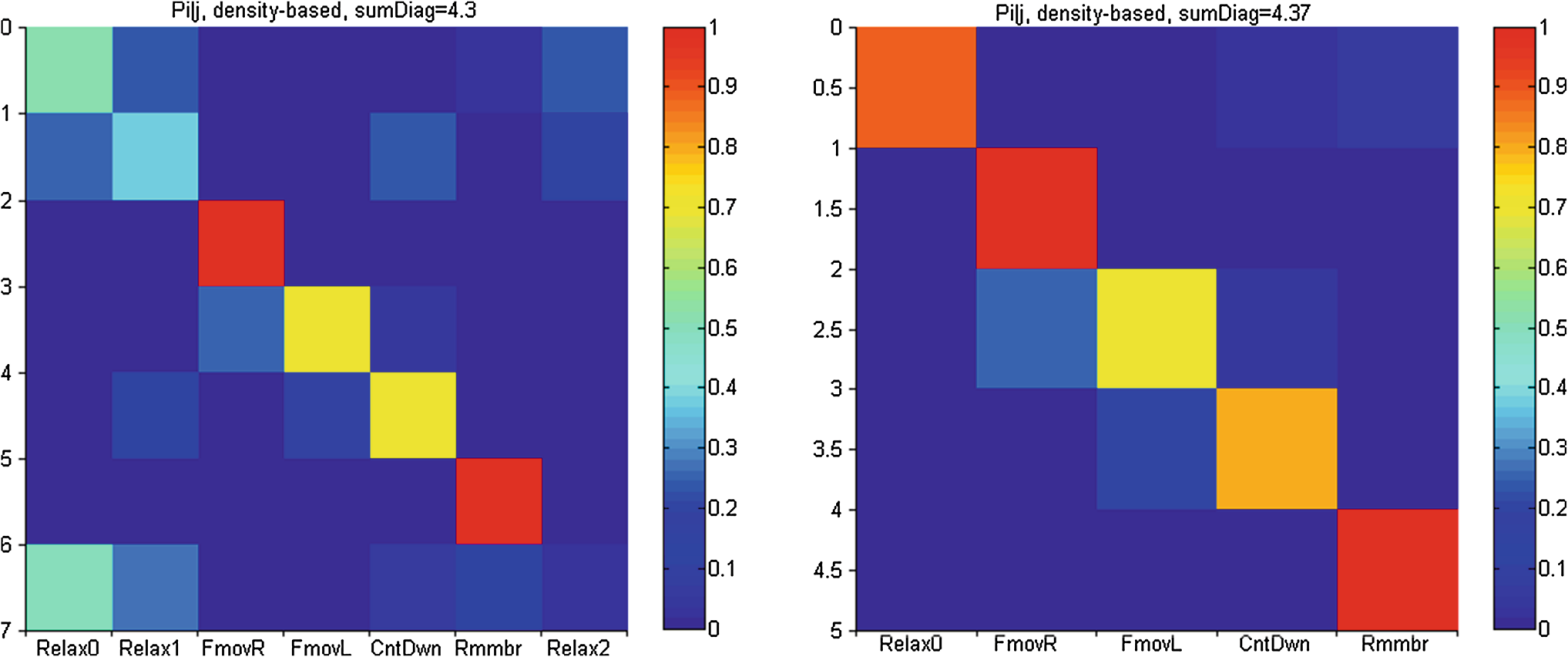
Specificity of the BAU-density maps. *Top-left*—depicts all seven tasks. There are mix-ups among the 3 relaxing periods (1st, 2nd, and 7th rows and columns), but there is good discrimination among the other tasks (FmovR, FmovL, CntDwn, and Rmmbr). The sum over the diagonal of this matrix was 4.3 (1 is random, 7 is perfect). *Top-right*—includes only Relax0 of the 3 relaxing periods. The sum over the diagonal was 4.37 (1 is random, 5 is perfect)

Are the maps specific to behavior? To answer this question, we split the data in each task into training and test sets and tested the similarity between each test set and the seven training sets, as described in the methods. This was repeated 100 times to obtain a confusion matrix (Fig. 7) showing the probability that a test set of task *i* is most similar to a training set of task *j*. If the maps are random, every cell in the matrix should be 1/7. If the maps are very specific, the main diagonal should be 1 and everything else should be zero. Thus, the sum of the diagonal is expected to be somewhere between 1 and 7.

The 3 relaxing tasks (Relax0, Relax1, and Relax2) exhibit partial internal confusion. Hence, during each of the relaxing states, also some specific mental processes are likely to have taken place. All three relaxing tasks were well-discriminated form the other tasks. When only one of the relaxing states (Relax0) was considered, the discrimination was very good (Fig. 7-right).

**Fig. 8:**
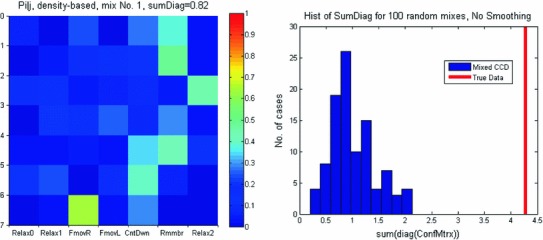
Confusion matrices for randomly shuffled data. Data for all 7 tasks (3 relaxing and 4 mental and motor tasks) were cut into 686 disjoint segments, mixed, and reassigned to 7 tasks so that each mixed task contained exactly 14 segments from each of the original tasks. This was repeated 100 times to obtain 100 sets, each with 7 mixed tasks. *Left*—the confusion matrix for mixture number 1. *Right*—histogram for the sum over the diagonal for all 100 mixtures. The *red line* is the sum for the unmixed data

When the confusion matrix was computed for data shuffled among all 7 tasks, the results appear random (Fig. 8-left). Random shuffling was done 100 times and the distribution of the sums over the diagonal of the confusion matrix was around 1, whereas for the true data, it was 4.3 (Fig. 8-right). Clearly, categorizing the task based on the density of BAUs over the hull was not driven by chance.

### Complex BAU-patterns

**Fig. 9:**
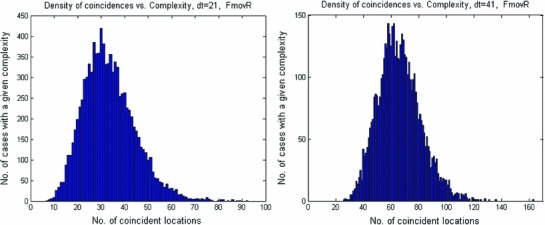
Distribution of coincidence-complexity. Data from FmovR (100 s.). Each event was spread over 21 (or 41) samples, and then, the point process was decimated 21 (or 41)-fold. *Left*—coincidences within 21 samples (10 ms). *Right*—coincidences within 41 samples (20 ms)

We predicted that some specific spatio-temporal BAU-patterns would be associated with every type of mental process. We started by studying coincidences. Our original time resolution was $\sim$0.5 ms. We extended this to 10 and 20 ms by convolving the point process of each location (consisting of many zeros and a few ones) with a box-car kernel of 21 or 41 ones. Then, we looked for coincidences among activities in the 532 parallel processes. To avoid multiplying the same coincidence over multiple samples (due to convolution), we decimated the data into steps of 21 or 41 samples and then looked for coincidences. Figure 9 illustrates the complexity of the coincidences (i.e., the number of locations participating in a coincident event).

As expected, there were a multitude of cases in which many locations in the brain had coincident BAUs within a short time. However, when examining their composition (which locations were coincident), we found that almost all occurred only once. The same held true for all the tasks studied here.

One explanation is that a kernel repeats many times, but each time, some other points in the brain also produce BAUs; hence, we do not see repetitions of the exact channel-composition of the coincidences. To examine this possibility, we tested for repeating coincidences of 5 locations (quintuplets) within 10 ms. When restricting the search to 4 or more repetitions of the same quintuplet, none were found. For 3 repetitions, none were found in 4 out of the 5 tasks but 7 quintuplets repeated 3 times each in the FmovL task.

**Fig. 10:**
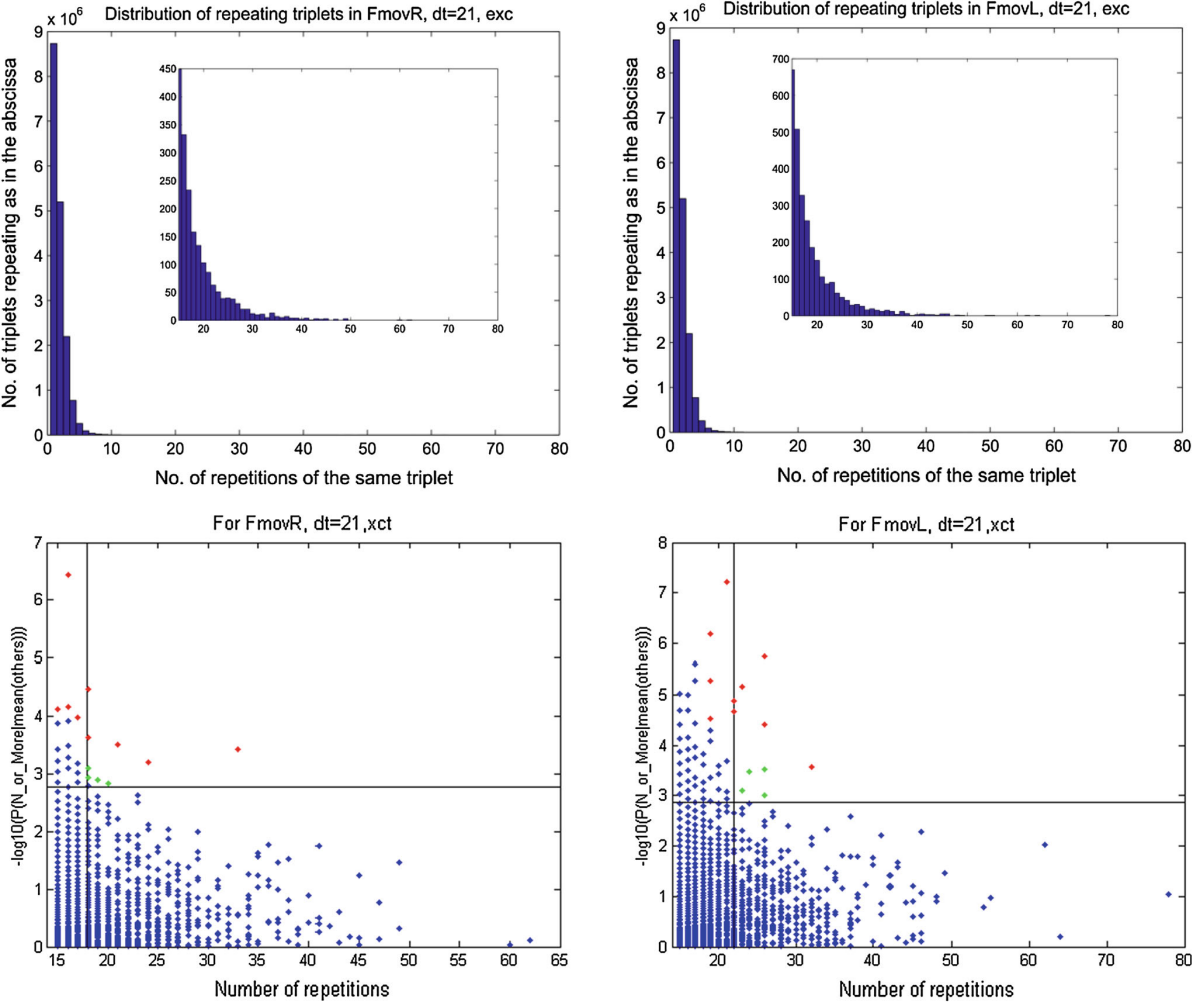
Repetitions of triplets. Data for tasks FmovR (and FmovL). Only triplet coincidences in 21 samples (10 ms) are considered. Out of 24,953,460 possible triplets, 17,320,986 (18,371,160) did occur. *Top*—the distribution of the number of times a certain triplet occurred. The *insets* show the tails of these histograms from 15 repetitions up. They included 1,896 (2,772) of the repeating triplets. *Bottom*—for each triplet, we computed the probability of having so many repetitions given the mean repetitions in the 4 other tasks. The cases plotted in red were considered as specific to the FmovR (FmovL) tasks. The ordinate is displayed in—$\hbox {log}_{10}$(probability)

However, if instead of repeating quintuplets we looked for repeating triplets, we found many triplets that repeated numerous times in each task, as shown in Fig. 10. The top two panels depict the distribution of repeating triplets for two tasks (FmovR and FmovL).

For each triplet repeating *N* times, we computed the probability of obtaining *N* or more repetitions given the mean number of repetitions of the very same triplet in the other 4 tasks (*M*) by

$$\begin{aligned} P\left\{ {N \hbox {or more}\left| {M =e^{-M}\sum \limits _{n=N}^\infty {{M^{n}}/{n!}} =1-e^{-M}\sum \limits _{n=0}^{N-1} {{M^{n}}/{n!}} } \right. } \right. \end{aligned}$$

We started the search for the 10 best triplets by looking for triplets that repeated more than 20 times and had a chance probability of repeating of $<$0.001. We shifted these two limits in steps of 0.5 % until 10 triplets were above both thresholds. The thresholds at this stage are shown as vertical and horizontal lines in Fig. 10-bottom part. Then, we selected the 5 points closest to the corner of the two thresholds and looked for a better replacement just to the left of the vertical threshold, but not below 15 repetitions. The green dots in Fig. 10-bottom were replaced by the red dots to the left of the vertical threshold line.

Did these 10 selected triplets occur by chance? Altogether, there were 1,896 (2,772) triplets that repeated more than 15 times. The expected number of instances with a probability of 0.001 is 1.896 (2.772). The probability of finding 10 or more cases when the expected number is so low is $<$0.00003 (0.0006). Thus, these 10 triplets are truly specific to the task. Very similar results were found for all the tasks.

#### Time precision of coordinated activity

When looking for patterns of order 5 or more that occurred within 5 ms and repeated at least twice, we found only one case in our entire data set. However, when increasing the time window from 5 to 50 ms, we found many quintuplets that repeated 33 or more times in each task. Altogether, 426 quintuplets from all 5 tasks were studied. How were the activities within each of these repeating quintuplets ordered, and what was the time precision of this order? This issue was studied by cross-correlating pairs of BAU-traces (BAUpp).

**Fig. 11:**
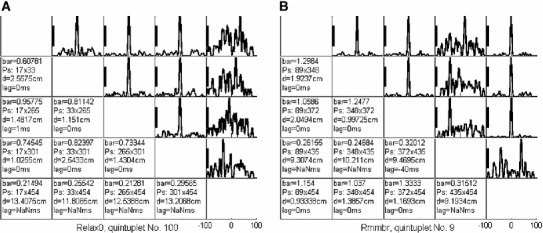
Cross-correlation table for all pairs of BAUpp within a quintuplet. For each trace of BAUpps taking part in a repeating quintuplet, the cross-correlation was computed by taking data around ($\pm 101\,\mathrm{ms}$) the occurrence of the quintuplet. Initially, the data had a 0.5 ms resolution and so did the cross-correlation. Then, the fine-grained correlation was smoothed with a Gaussian bin (std = 2 ms). All possible pair-wise combinations are illustrated. The main diagonal is the autocorrelation with an extremely high peak at 0 by definition. Autocorrelations are not illustrated. The graph in panel (*i*,*j*) is by definition the time- reversed version of the graph in panel (*j*,*i*); therefore, only the upper diagonal is illustrated. In its symmetrical position, the following data from *top to bottom* are provided: The size of the calibration bar; the Id’s of the two BAUpps, the distance between them, the lag of the peak (NaNms indicates no large enough peak was detected). Only peaks that were at least twice as high as any other peak were considered. The timescale for the abscissa is shown under the *lower-right box*. **a** For a quintuplet that repeated 39 times during 100 s of the Relax0 task. The situation here, with 4 traces with a high near the 0 lag correlation, is the most typical situation. **b** For a case also including long delays. Data taken from task Rmmbr and a quintuplet that repeated 40 times. Four BAUpps showed a tight correlation near 0 lag. Some of them also had a tight (though weaker) correlation with the $4\mathrm{th}$ BAUpp at long ($-$40 ms) delays (as seen in the fourth column)

For every pair of BAUpps within a quintuplet, we extracted the data within $\pm 101\,\hbox {ms}$ around its time of occurrence and computed the cross-correlation (10 cross-correlations per quintuplet). Figure 11 shows the cross-correlations between all pairs for one quintuplet. Of 4,260 cross-correlations (10 for each quintuplet), 2,661 showed a clear sharp peak. Most of these (2,529) had their peak within $\pm 2\,\hbox {ms}$. Only 21 had a lag of 2–10 ms, and 11 had a lag above 10 ms. An example of a tight correlation with a long lag is illustrated in Fig. 11b.

Many of the channels with tight cross-correlation were next neighbors (distance of $\sim$8 mm), but not all. For example, the cross-correlation shown in the top-left panel of Fig. 11a was between traces at two points 2.5 cm apart. The long lag in Fig. 11b was between points over 9 cm apart.

## Discussion

The initial motivation for this research was the observation that when synfire chains were simulated and two of them in different regions became synchronized, the total activity in each region was initially increased and then reduced to a little above the background level (Hayon et al. 2004, 2005; Aviel et al. 2003). With this observation, it seemed logical to test whether such processes may be observed in macroscopic measurements of cortical activities. However, due to the organization of cortical inhibition (being local) and of cortical excitation (being both local and among areas), brief increases in activities may be the result of interactions between other types of networks. Thus, the formulation of our hypotheses is more general:

1. Mental activities are generated by coordinated activity in many brain regions.
2. When two regions start to coordinate their activity, there is a brief increase in activity in both.
3. Such brief increases may be detected in recordings of electrical activity of a cortical region.
4. When the same mental task is repeated frequently, the same regions become coordinated in the same manner.

We found that BAUs can be found in CCDs as extracted by our octahedron method (See Lots et al. 2013, for a further analysis of this method). Figure 5 shows that during such a BAU, there is a major rearrangement of cortical activity as manifested by the threefold to fourfold decrease in the variance around these BAUs.

Furthermore, we found that the map of densities of the BAUs over the cortex may be reliably used to distinguish between the various mental tasks employed here (Fig. 7). Thus, the BAUs show some specificity with regard to behavior and cannot be regarded as chance events. These findings support hypotheses 2 and 3.

We found numerous instances where many cortical locations showed coincident activity within 10 (or 20) ms (Fig. 9), which is in line with our first hypothesis. However, almost all of these coincidences repeated only once. Even quintuplets did not repeat more than 3 times unless a wider (50 ms) window was permitted. This runs counter the fourth hypothesis.

Several explanations can be put forward. The behaviors in our task were not truly repetitive. During relaxation or while recalling a movie, there is no reason why the same mental process will repeat again and again. Finger movements had repetitive components, but the subject invented a new sequence every few repetitions. So there was no repetition throughout the 100 s of recording. The mental arithmetic task needed repetitive ‘calls’ for the subtracting algorithm, yet subtracting 7 from 2007 is much simpler than from 3001. This situation calls for repeating this type of analysis where there are repetitive mental processes by construction. Such experiments are now under way.

It is also possible that in parallel with the conscious processes during a directed mental task, multitude of sub-conscious processes take place. Each of these involves its own specific BAUs. The detected coincidences may then be a mixture of task-specific BAUs with many other, unrelated, BAUs masking the purely task-specific ones.

Second, we may have been too permissive in detecting BAUs, and stricter criteria could have led to better results. The localization of CCDs may not have been sufficiently accurate, and we should have merged activities in neighboring locations. If a BAU appeared at times in one location and at others in its neighboring location, this could reduce the number of repetitions detected.

On top of the above technical issues, an alternative that must be considered is that the fourth hypothesis is wrong. The same mental process can take many routes through the cortex, such that if studied with detailed spatial and temporal resolution, it is likely that we would almost never see an exact repetition of the same pattern of activation. Clearly, the second time we see a picture is definitely not the same as the first time. This scenario is reminiscent of Edelman’s (1978) re-entrant hypothesis. If so, this calls for a paradigm shift in our studies of the electrical manifestations of mental activities.

Nevertheless, we have presented a useful way to study the dynamics of brain activity that is not dependent on averaging over time or frequencies and therefore can reveal brain processes instantaneously.
